# The association of school-related active travel and active after-school clubs with children’s physical activity: a cross-sectional study in 11-year-old UK children

**DOI:** 10.1186/s12966-019-0832-3

**Published:** 2019-08-22

**Authors:** Ruth Salway, Lydia Emm-Collison, Simon J. Sebire, Janice L. Thompson, Deborah A. Lawlor, Russell Jago

**Affiliations:** 1 Centre for Exercise, Nutrition & Health Sciences, School for Policy Studies, University of Bristol, 8 Priory Road, Bristol, BS8 1TZ UK; 2 School of Sport, Exercise and Rehabilitation Sciences, University of Birmingham, Birmingham, B15 2TT UK; 3 MRC Integrative Epidemiology Unit at the University of Bristol, Oakfield House, Oakfield Grove, Bristol, BS8 2BN UK; 4 Population Health Sciences, Bristol Medical School, University of Bristol, Canynge Hall, Whiteladies Road, Bristol, BS8 2PS UK

## Abstract

**Background:**

Physical activity is associated with improved physical and mental health among children, but many children do not meet the recommended hour per day of moderate-to-vigorous-intensity physical activity (MVPA). The aim of this paper is to investigate participation in active after-school clubs and active travel to and from school at age 11 and estimate the average daily minutes of MVPA associated with active club attendance and active travel.

**Methods:**

Accelerometer data were collected on three weekdays for 1296 11-year-old children in a cross-sectional study. Children reported attendance at active after-school clubs and how they travelled to and from school for each day of the week. To account for repeat days within child and clustering within schools we used multilevel models with random effects at the school and child level, and fixed effects for all covariates. We calculated odds ratios for participation in active after-school clubs and active travel for gender, measures of socio-economic position and BMI category. We also explored the association between active club attendance, active travel and daily average MVPA.

**Results:**

Boys and girls were equally likely to attend active after-school clubs. Boys were more likely to travel to school using active modes. Attendance at active after-school clubs and active travel home were not associated with each other. Attending an active after-school club was associated with an additional 7.6 min (95% CI: 5.0 to 10.3) average MVPA on that day among both boys and girls. Active travel was associated with an additional 4.7 min (95% CI: 2.9 to 6.5) average MVPA per journey for boys and 2.4 min (95% CI: 1.0 to 3.7) for girls.

**Conclusions:**

Both active after-school clubs and active travel are associated with greater physical activity on the day that children participate in these, and we saw no evidence that those attending active clubs do so at the expense of active travel home afterwards. While the increased daily MVPA is small to moderate, active after-school clubs and active travel on multiple days of the week could make important contributions as part of complex interventions aimed at increasing population levels of physical activity in children.

**Electronic supplementary material:**

The online version of this article (10.1186/s12966-019-0832-3) contains supplementary material, which is available to authorized users.

## Background

Physical activity in children is associated with reduced risk of obesity and improved emotional well-being in childhood [1], as well as lower levels of a number of risk factors for cardiovascular disease and type 2 diabetes [2]. It is recommended that all children and young people should engage in an average of an hour per day of moderate-to-vigorous-intensity physical activity (MVPA) [3] but many children do not meet these guidelines, with girls less active than boys at all ages [4, 5].

Most interventions aimed at increasing children’s physical activity have been delivered during school time [6, 7]. However, schools often face difficulty in timetabling additional physical activity sessions, and so there is a need to understand the role of school-orientated physical activity outside of the curriculum. The after-school period has been described as ‘critical hours’ for young people’s physical activity as it is a period of time when children can decide if, and how, they are to be active [8]. Data from the Avon Longitudinal Study of Parents and Children showed that the highest peaks of physical activity within a day occurred during the after-school time period [9]. There is therefore a need to explore different options for physical activity during this time, such as extra-curricular programmes and active travel, and how they contribute to activity levels.

Physical activity-based clubs in a school setting outside of the curriculum (‘active after-school clubs’) have the potential to facilitate physical activity for children, by utilising school space and often school staff. Current provision of active after-school clubs in the UK is high, with 91% of schools offering at least one, and on average 4.8 active clubs a week, to pupils aged 9–11 years, with the majority being team-based sports [10]. There is little information on the current levels of participation in active after-school clubs, although previous data from our study [11] found higher participation among 8–9 year old boys than girls. While several studies have explored the potential for increasing children’s physical activity via after-school programmes, evidence for the effectiveness of such interventions is mixed and shows considerable variation [8, 12–14]. In particular, some studies suggest that boys may gain more MVPA benefit than girls [15, 16], but evidence of a gender difference is inconclusive. Moreover, without information on participation in such programmes, it is difficult to say how they could affect physical activity in the general population.

Active travel to and from school is an important source of regular, daily physical activity for children. In the UK the proportion of primary school children walking to school has remained around 50% between 2002 and 2017 [17], whereas in the US some studies suggest that rates of active travel may be declining [18]. In addition, those who actively travel to school are 30% more likely to actively travel to other destinations [19]. Active travel is associated with increased MVPA but not lower body weight [20], and while some studies have found differences in the benefits of active travel between boys and girls, again the evidence is inconclusive.

Active after-school clubs and active travel home from school are potentially competing activities in the after-school period, and it is possible that any increase in MVPA for a child who attends an active after-school club is offset by then not actively travelling home, for example due to the change in their usual arrangements [21]. It is therefore important to understand the interplay between active travel and after-school club attendance in contributing to daily physical activity. Furthermore, understanding who is currently participating in each type of activity may help in targeting interventions or messaging to specific subgroups. In this paper, we analyse daily data rather than aggregating across the week. The main benefit of this is being able to reduce within-child variability and compare the MVPA on a day with an active club and/or active travel, compared to a day without for the same child. It also allows us to explicitly explore whether an active club after school replaces or supplements active travel home. The aim of this paper is to investigate participation in active after-school clubs and active travel at age 10–11, and whether there is any relationship between the two after-school activities. In addition, we look at the association of club attendance and active travel with daily minutes of MVPA.

## Methods

Data are from the B-PROACT1V study, a longitudinal study that examined the physical activity and sedentary behaviours of primary school children aged 5–11 years, and their parents [5, 11, 22]. This paper uses cross-sectional data collected between March 2017 and May 2018 from Phase 3 of the study, when the children were aged 10–11 years, and comprises 1296 participants from 50 schools in the southwest of England. Of these, 708 participated at baseline in 2012/13, and 588 were new participants. The study received ethical approval from the School of Policy Studies Ethics Committee at the University of Bristol, UK, and written parental consent was received for all participants [23].

### Accelerometer data

Children wore a waist-worn ActiGraph wGT3X-BT accelerometer for three weekdays and two weekend days. Accelerometer data were processed using Kinesoft (v3.3.75; Kinesoft, Saskatchewan, Canada) and analysis was restricted to those children who provided at least 1 day of valid weekday data. A valid day was defined as at least 500 min of data, after excluding intervals of ≥60 min of zero counts allowing up to 2 min of interruptions [4]. Data were recorded at 10 s intervals and characterised as sedentary, light or MVPA using Evenson population-specific cut points for children [24]. The average number of MVPA minutes for each weekday were derived for each child, and we recorded the number of minutes the accelerometer was worn on each day. We also derived the average weekend MVPA for each child to use as a proxy for non-school day physical activity levels, as using daily levels of MVPA is likely to be influenced by participation in active after-school clubs and active travel.

### Active after-school clubs and travel

Children completed a questionnaire at the time accelerometers were issued, and were asked, for each day of the week, whether they typically attended an after-school club at their school focussed on playing a sport or being active (excluding general after-school childcare clubs). These responses were coded as ‘Yes’ or ‘No’ for each day of the week. In addition, they were asked how they travelled to and from school for the main part of their journey on each day of the previous week, with options walk, bicycle, scooter, car or public transport. These were coded as either active travel (walk, bicycle or scooter) or non-active travel (car or public transport).

### Other measures

Child height and weight were recorded to the nearest 0.1 cm and 0.1 kg by trained fieldworkers, and body mass index (BMI) was calculated and converted to an age- and sex-specific standard deviation score based on UK reference curves [25, 26]. These were used to create age- and sex-specific overweight and obesity indicators using 85th and 95th percentiles, respectively. As less than 2% of children were underweight, we did not analyse them as a separate category but included them in the healthy weight category.

As different indicators of socio-economic position measure different, but often related, aspects [27, 28], we included two such measures: household education, which provides an indirect indication of income, and the index of multiple deprivation (IMD) for the neighbourhood in which the child lived, an area-based measure which captures the socio-economic conditions of the area. An adult in the household was asked the highest education qualification of anyone in the household, with categories ‘Up to GCSE/ O level or equivalent’ (UK qualification usually acquired at age 16), ‘A level/NVQ or equivalent’ (qualification usually acquired at age 18), ‘University Degree/HND or equivalent’ and ‘Higher Degree (MSc/PhD) or equivalent’. Where education data were missing (*n* = 215 (17%)), we used a response from earlier phases of the project if available. In 32 cases the response from Phase 1 was used, in which the respondent (mostly mothers) was asked for their own education rather than the highest in the household and so highest household education may be underestimated for these respondents. Indices of Multiple Deprivation (IMD) scores, based on the English Indices of Deprivation (http://data.gov.uk/ dataset/index-of-multiple-deprivation), were assigned to each child based on their reported home postcode. Higher IMD scores indicate a greater level of deprivation.

### Statistical analysis

We compared child characteristics in the sample to baseline measurements from Phase 1 at age 6. Child characteristics and overall participation in active after-school clubs and active travel were summarised. Average MVPA, active after-school clubs and active travel were additionally summarised by day, and the different modes of transport described.

Analysis was conducted at the daily level to identify associations of clubs and travel with MVPA on the days on which the activities occurred, so each child contributed data for a maximum of 3 days across the school week. This also allowed us to explore whether those who attended an active after-school club were less likely to then actively travel home on that day. The three weekdays of data collection depended on school availability, which is unlikely to be associated with children’s MVPA, active clubs or active travel. Data were available for every day of the week with slightly more on Mondays and Fridays as the study also collected weekend data. We compared participation in active clubs and travel between days with and without accelerometer measurements, to see if there were any differences due to collecting only three out of a possible five weekdays.

To account for repeat days within child and clustering within schools we used multilevel models with random effects at the school and child level, and fixed effects for all covariates. We used logistic multilevel models to calculate odds ratios for participation in active after-school clubs and active travel by child characteristics (gender, IMD, household education and BMI) and by weekend MVPA, as an indication of how active a child generally is. BMI associations were adjusted for confounders gender, education and IMD. For active travel, we additionally estimated the odds ratio for active travel home from school after an after-school club by including active club as a covariate in the model, adjusting for gender, household education, IMD, BMI category and average weekend MVPA to see if an active club was replacing rather than supplementing active travel. A negative coefficient for the active club variable means that active travel is less likely on a day with a club. We fitted models for active travel both to and from school, but as the large majority of children (85%) use the same mode of travel for both journeys in a day, we have reported results for active travel home from school only.

**Fig. 1:**
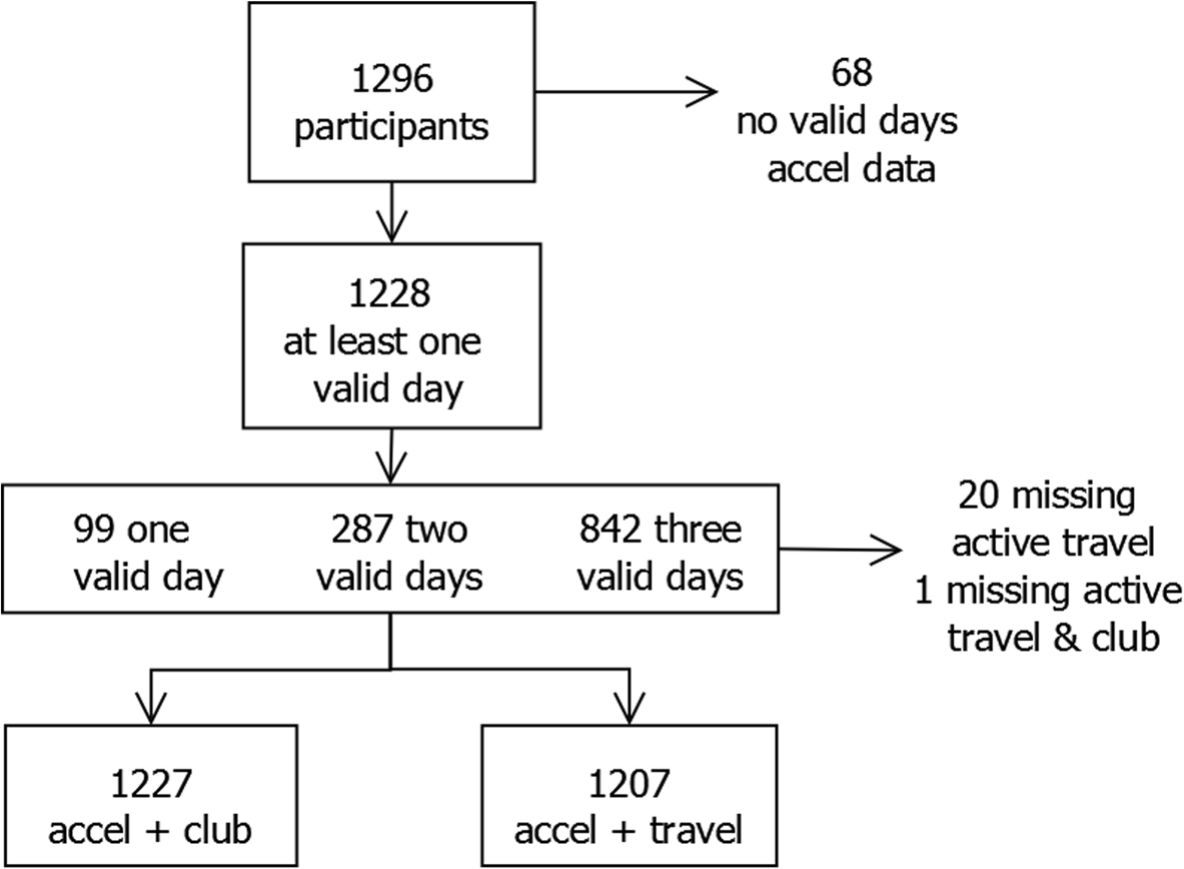
Flow diagram of participants and missing data. *All 1207 participants with accelerometer and active travel data additionally have active club data

To explore the association between active clubs and travel and daily average MVPA, we used linear multilevel models adjusted for accelerometer wear time, day of the week, gender, BMI category, household education and IMD. To explore possible gender differences, we fitted models separately for girls and boys and ran models with both genders included with an interaction term between gender and participation. We included all children who had valid accelerometer data on at least 1 day (*n* = 1228; 5% excluded), with a maximum of 3 days (65%), in the multilevel models, under a missing at random assumption, which amounted to 3206 days with measurements (Fig. 1). Due to the way the questionnaire was administered, there was very little missing active club and travel data (2%) for these children. All analyses were performed in Stata V.15.0.29 [29] and MLwiN [30] within Stata, via the runmlwin command [31].

## Results

The sample was broadly representative of the baseline study in terms of gender, age and SEP, with slightly fewer children who were obese at age 6 (Additional file 1: Table S1). There were no differences in active club attendance or active travel between days included in the analysis when children provided accelerometer data, and days when they did not (Additional file 1: Table S2).

### Participant characteristics

Table 1 summarises the characteristics of the participants. Over half (57%) of children attended no active after-school clubs, with an average of 0.7 clubs per week per child. Average attendance for those who attended at least one active after-school club was 1.7 days per week. Active travel was consistent across the week, with 55% of children travelling either to or from school on all 5 days of the week while 24% never used active travel. Table 2 reports the prevalence of active travel to and from school, participation in active after-school clubs and daily average MVPA by day. MVPA varied across the week, with children more active in the second half of the week. Active travel did not vary across the week, and journeys were consistent, with the majority of children undertaking active journeys on all or no days (Table 1). Active club attendance varied across the week, with higher attendance mid-week. The most common forms of travel were walking (47% of all journeys) and by car (39%), with far fewer using scooters (7%), bicycles (5%) or public transport (2%).

**Table 1:** Characteristics of sample

	Mean (Sd) or %	Missing N (%)
% female	52%	0 (0%)
Age (years)	10.9 (0.4)	110 (8%)
BMI z-score	0.35 (1.16)	11 (1%)
BMI category		
Healthy weight	71%	
Overweight	14%	
Obese	15%	
Household education		105 (8%)
GCSE	20%	
A level	26%	
University Degree	37%	
Higher Degree	17%	
IMD score	15.4 (14.4)	45 (3%)
Average weekday MVPA	60.4 (23.3)	69 (5%)
% with 1 weekday	8%	
% with 2 weekdays	22%	
% with 3 weekdays	65%	
Number of active clubs (per week)		2 (< 0.5%)
None	57%	
One per week	24%	
Two or more per week	19%	
Mean active clubs (per week)	0.7 (1.1)	
Mean active clubs among those who do any active clubs (per week)	1.7 (1.0)	
Active travel days^a^ (per week)		25 (2%)
None	24%	
1–2 days	8%	
3–4 days	12%	
5 days	55%	
Mean active travel days^a^ (per week)	3.3 (2.1)	

^a^Days on which at least one journey is active travel

**Table 2:** Active after-school clubs, active travel and average daily MVPA by day

	Mon	Tue	Wed	Thu	Fri	Mean (sd)
N	694	486	548	595	883	1228
Active club	13%	15%	17%	16%	12%	0.7 (1.1)
Active travel to school	58%	58%	58%	57%	58%	2.9 (2.2)
Active travel from school	60%	61%	60%	59%	59%	3.0 (2.2)
No. with accel data	694	486	548	595	883	
Mean MVPA^a^ (min)	53.7	56.1	66.9	62.7	63.4	60.4 (23.3)
(sd)	(26.6)	(25.7)	(33.5)	(27.1)	(28.3)	

^a^Mean of the average daily MVPA

### Participation in active clubs and travel

**Fig. 2:**
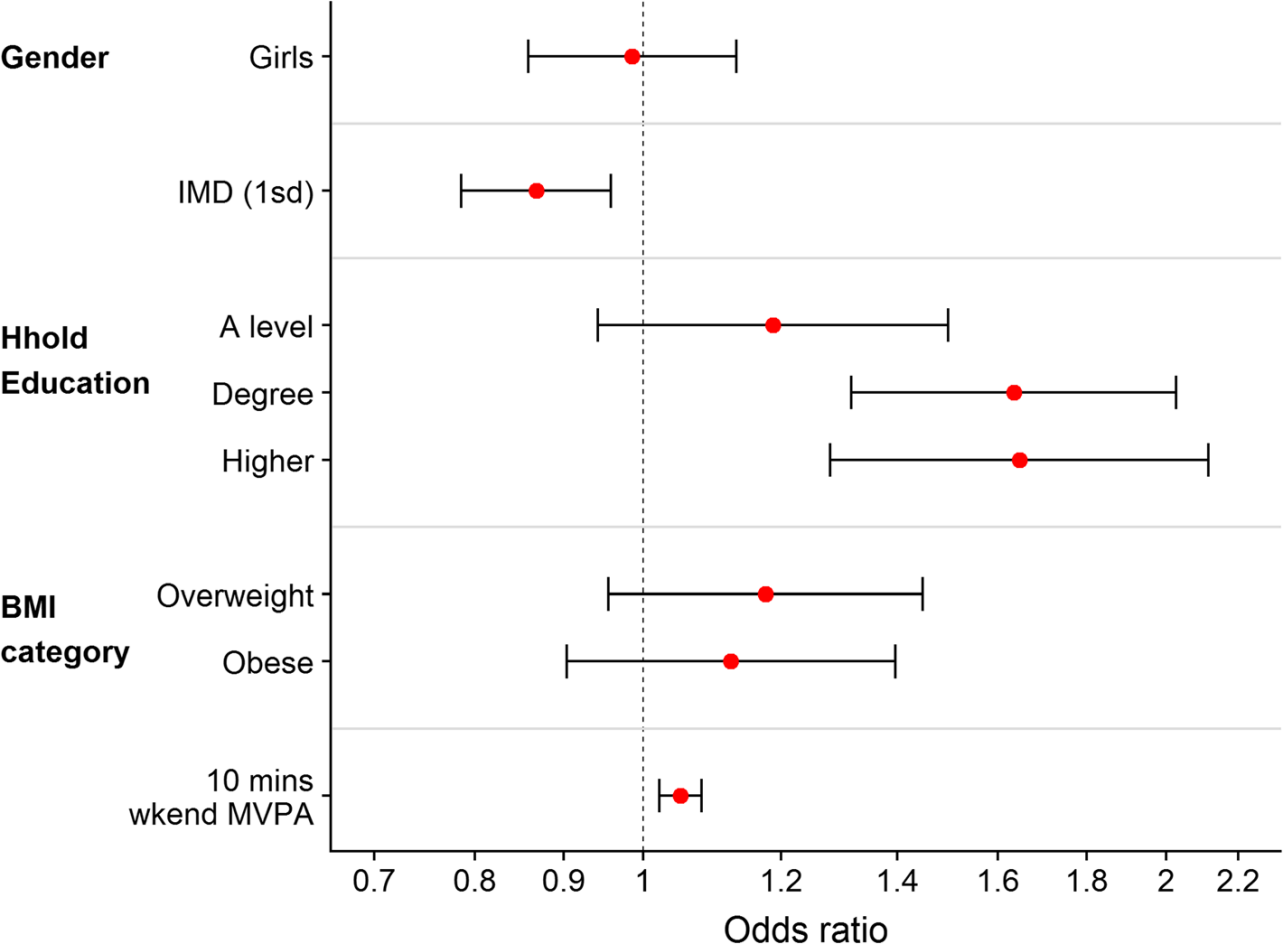
Odds Ratios for participation in active after-school clubs. Reference categories: Boys (Gender), Up to GCSE (Household education) and Healthy weight (BMI category). Continuous variables: odds ratio for an increase of 10 mins in weekend MVPA, and an increase of 1 S.D. (14.4) in IMD (higher IMD score is more deprived)

**Fig. 3:**
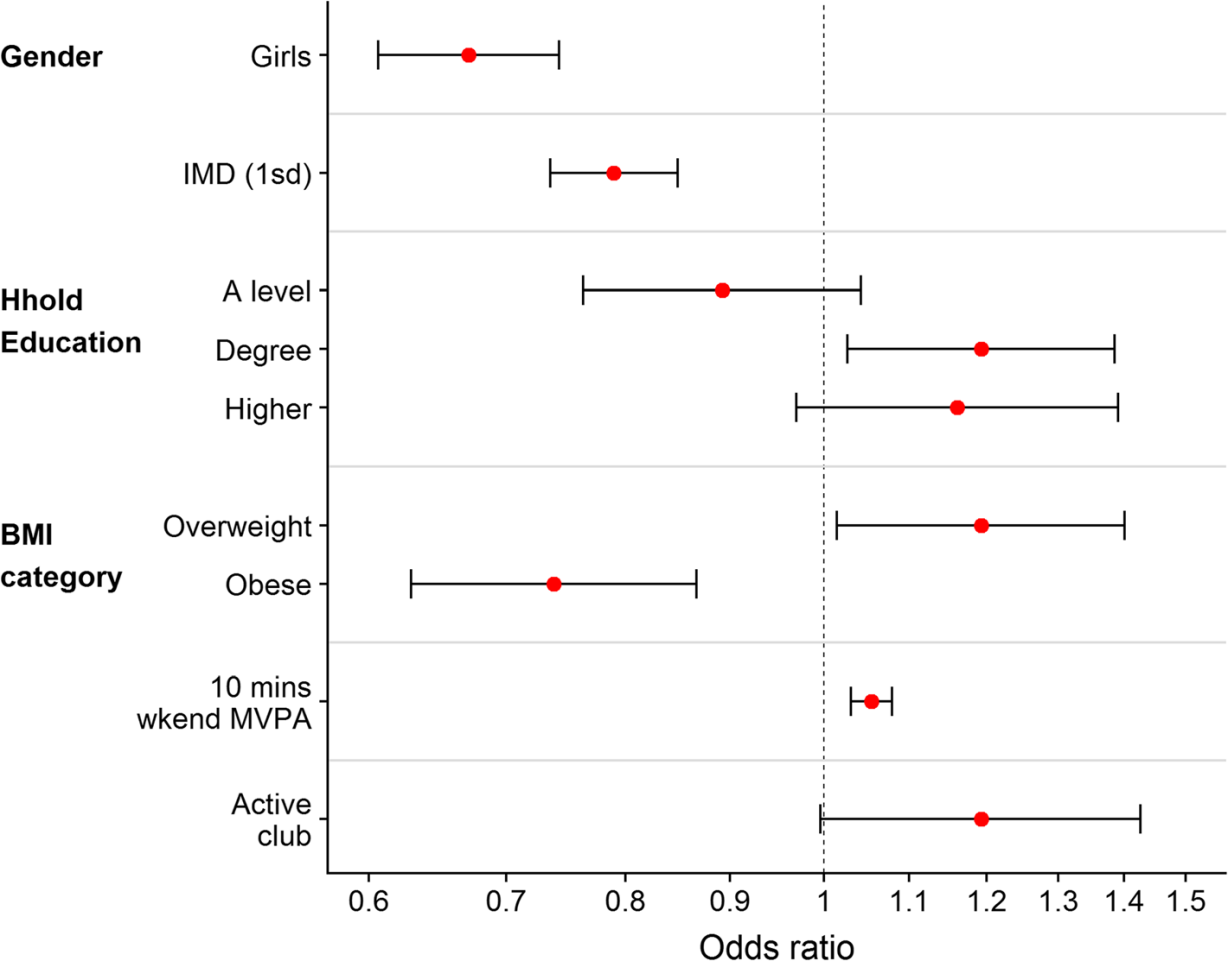
Odds Ratios for participation in active travel (from school). Reference categories: Boys (Gender), Up to GCSE (Household education), Healthy weight (BMI category) and No attendance at active club (Active club). Continuous variables: odds ratio for an increase of 10 mins in weekend MVPA, and an increase of 1 S.D. (14.4) in IMD (higher IMD score is more deprived)

Figures 2 and 3 (see also Additional file 1: Tables S3 and S4) summarise the odds ratios for factors associated with participation in active after-school clubs and active travel from school respectively. Both boys and girls were equally likely to attend active after-school clubs, and there was no difference in participation by BMI category. Both measures of SEP were associated with club participation, with those living in less deprived areas and those from households with higher educational qualifications more likely to attend, with the odds of attending a club for those from households where the highest qualification was a University Degree 63% higher than those from GCSE-qualified households. Children who were generally more active, as defined by higher weekend MVPA levels, were slightly more likely to attend an active club: every 10 min extra average weekend MVPA was associated with a 5% increase in the odds of attending a club. Patterns for active travel were different, with the odds of using active travel 49% higher among boys than girls. Those living in more deprived areas (higher IMD) were less likely to use active travel, while those from households with higher educational qualifications were more likely to use active travel. We saw differences in active travel by BMI category, with those who were obese were much less likely to use active travel, although those who were overweight were slightly more likely to use active modes of travel. Those who were generally more active were more likely to use active travel. Children who attended active after-school clubs were no less likely to actively travel home than those who did not. The results reported here are for active travel from school; on 87% of days children who used active travel from school also used active travel to school, and the odds ratios and patterns were similar for the association of participation in active travel to school (results available from corresponding author on request).

### MVPA associated with active clubs and travel

Attending an active after-school club was associated with an additional 7.6 min average MVPA on that day for all children, compared to those not attending (Table 3). The separate models by gender suggest that boys gained around 3 min more MVPA from active after-school clubs than girls, but the large variability means the importance of this differences requires further examination in other datasets. Active travel was associated with an increase of 3.4 min per journey in average MVPA on that day for all children, but gender differences were more marked. A boy who travelled both to and from school using active travel had an average of 9.4 min (95% CI: 5.8 to 13.0) higher daily MVPA than a boy who did not actively travel, whereas for a girl this was an average of 4.7 min (95% CI: 2.0 to 7.4) higher daily MVPA.

**Table 3:** Difference in average daily MVPA on days with active travel/clubs compared to days without

	Difference in MVPA (min)	95% CI	*p*-value
Active club (per club)			
All	7.64	(5.00, 10.28)	
Boys	9.15	(4.87, 13.43)	
Girls	6.49	(3.26, 9.73)	
Test for gender interaction			0.223
Active travel (per journey)			
All	3.42	(2.31, 4.53)	
Boys	4.70	(2.89, 6.52)	
Girls	2.35	(1.01, 3.69)	
Test for gender interaction			0.028

All models adjusted for day of week, wear time, gender, BMI category, household education and IMD

## Discussion

### Participation in active clubs and travel

Just over half of children attended an active after-school club at least once a week. We found no difference in active club participation between boys and girls, which is in contrast to our earlier results from Phase 2 of the study [11]. This is in part because previously we reported on all school clubs, including those within school time, but may also reflect a difference in either availability or uptake in active clubs between Year 4 (age 8–9) and Year 6 (age 10–11). In addition, there were no differences in participation by BMI category. This suggests that the provision of school-based extracurricular active clubs is a valuable addition to equalising participation among groups who are typically less active. However, children from more deprived areas and from households with lower educational qualifications were less likely to attend clubs. This may reflect an income disparity, since around a third of after-school clubs are paid for by parents [10]. Thus, there is a need to ensure that these opportunities are accessible to everyone, especially considering their ability to reach those who are less active.

Active travel participation was high in our study, with around 67% of children using active modes of travel on 3 or more days per week. This is higher than the national estimate of 51% for primary school children (ages 5–10) from the National Travel Survey 2017 [17] and may reflect that the children in the current study are at the top of the age range in the National Travel Survey. Boys were around 50% more likely to use active travel than girls. At an age when children who walk to or from school are more likely to be unaccompanied by parents, this may reflect safety concerns especially among parents of girls. Active travel was associated with deprivation, with lower levels of active travel in more deprived areas, which may reflect characteristics such as distance to school, traffic safety and perceptions of the environment [32]. As active travel patterns seemed to be stable across the week, replacing non-active modes of travel with more active methods could have cumulative effects across the week. One area of interest was whether those attending active clubs might do so at the expense of walking home. We saw no evidence of such displacement, indeed, those who attended active after school clubs were slightly more likely to also travel home actively. This suggests that the benefits of active after-school clubs and active travel may be considered cumulative.

We found that being more active generally (using higher average weekend MVPA as rough indicator) was associated with slightly higher participation in both active after-school clubs and active travel. It is not clear if children are more active because they do these activities, or they choose to do them because they are more active.

### MVPA associated with active clubs and travel

We found the benefits of active after-school clubs and active travel to be small to moderate in size, with a daily average MVPA of 7.6 min associated with an active after-school club and 3.4 min per active travel journey on that day. Replacing 10 min per day of sedentary time with MVPA has been associated with improved cardiometabolic indicators [33] and our findings are comparable to those levels found in behaviour change interventions [14]. It is important to recognise that the relationship between activities such as active clubs and travel, physical activity levels and health are part of a complex system [34] and thus while individual behaviour changes may not be effective alone, they may contribute to a complex intervention along with other elements that results in overall increases in physical activity at a population level. Thus, while our observed increase of 5–8 min MVPA on a single day is modest in isolation, it may form an important contribution when combined with other behaviour changes of a similar size.

We found that girls received less benefit than boys from active travel, which could reflect distance, walking speed or other behaviour during the journey. It is less clear whether there is a gender difference in MVPA for active after-school clubs, and previous evidence has been likewise mixed [14]. This could reflect difference choices of activities, although one previous study [16] found that boys accumulated significantly more MVPA compared with girls enrolled in the same after-school programme. These potential gender differences highlight the importance of engaging girls as a priority for future work [35].

### Implications

Our analysis examined active clubs and travel on a daily basis, rather than averaging MVPA across the week, which reduces within-child variability. Physical activity guidelines are based on average weekly MVPA minutes, so for comparability with other studies we must consider whether these small daily increases are meaningful when considering average MVPA across the whole week. Previous work has highlighted important differences between physical activity patterns on weekdays and weekends [36], and this paper only considers weekday activity. The observed differences in MVPA are based on a maximum of three weekdays of data per child so we must be cautious in extrapolating to the full week, although weekdays with and without data were very similar in terms of MVPA and participation in active clubs and travel. Over half of children did not attend any clubs and those that did attended an average of 1.7 clubs a week, with an MVPA increase of 7.6 min associated with club attendance. Active travel, while offering smaller individual MVPA benefits of 4.7 min for boys (2.4 min for girls) per journey, are more common and those that actively travel tend to do so all 5 days of the week. Thus, for those children, this represents a steady contribution on weekdays. While we cannot assume that a child who currently engages in no active travel would necessarily gain the same levels of MVPA if they were to move to active modes of travel, active travel may offer the opportunity of small amounts of regular physical activity, especially for those who are typically less active.

School-orientated activity outside of the curriculum offers an opportunity for schools to support and encourage active behaviour without the need to compromise curriculum time. Active clubs offer opportunities to engage those who tend to be less active and may be popular because of the ease of access via the existing school setting [15]. In contrast, active travel offers the opportunity for small regular sessions of physical activity that accumulate across the week, without the need for a formal active setting. Children participate in each of these activities depending on a combination of their personal preference and family or external circumstances, and it is encouraging to see that clubs are in addition to, rather than replacing active travel. While circumstances may not always allow both activities, currently just under half of children attend no active after-school clubs, and 20% of children use active modes of travel on some days but not others. Thus, it may be beneficial to investigate ways to encourage participation in existing active clubs, as well as to support increased active travel among those for whom it is feasible, with specific efforts to increase girls’ participation.

### Strengths and limitations

A strength of this study is the ability to analyse the data at a daily level within children, to link active after-school clubs, active travel and daily MVPA directly on the days they are undertaken. This also avoids any attenuation effect of aggregating MVPA and clubs/travel over the week, which can lead to underestimating the important contributions of these activities to meeting PA guidelines. A limitation of this choice is that it is susceptible to between-day variability in MVPA, although we have accounted for this to some extent by including day of week in our models. As child questionnaires were administered at the same time as the accelerometers were issued, nearly all children with valid accelerometer data also have data on active travel and clubs (98%) so there is very little missing data. Although we are limited in having only three out of five weekdays of accelerometer data per child, the data collection process means that the 3 days were allocated to schools effectively at random, and we saw no differences in active clubs or travel between days with and without accelerometer data. We asked the child how they travelled to school in the week prior to the accelerometer data, and so this might dilute any association if either week were atypical. We have cautiously interpreted average weekend MVPA as an indication of how active a child generally is, but differences between weekday and weekend levels of activity mean that this may not always be representative. In addition, as this is a cross-sectional analysis it is not possible to establish the direction of the association between participation on activities and being active. Finally, data are from a single region in the UK and so may not generalise to other settings.

## Conclusions

Both active after-school clubs and active travel are associated with greater physical activity on the day that children participate in these, and we saw no evidence those attending active clubs do so at the expense of active travel home afterwards. Active after-school clubs and active travel could make important contributions to levels of physical activity if boys and girls took part. While the increased daily MVPA is small to moderate, active after-school clubs and active travel on multiple days of the week could make important contributions as part of complex interventions aimed at increasing population levels of physical activity in children.

## Additional file

Additional file 1: **Table S1.** Comparison of sample characteristics with baseline. **Table S2.** Comparison of days with and without accelerometer measurements. **Table S3.** Associations of gender, SEP and BMI with participation in active after-school clubs. **Table S4.** Associations of gender, SEP and BMI with participation in active travel (from school). (DOCX 19 kb)

## Data Availability

The datasets generated during the current study are not publicly available due as the project is ongoing and data are not ready for archiving. We will consider reasonable requests for access to the data after the project is complete in 2020.

## Abbreviations

BMI: Body-mass index
IMD: Indices of Multiple Deprivation
MVPA: Moderate-to-vigorous-intensity physical activity
OR: Odds ratio

## References

[1] Strong WB, Robert M, Malina RM, CJR B, Daniels SR, Dishman RK, Gutin B, Hergenroeder AC, Must A, Nixon PA, Pivarnik JM (2005). Evidence based physical activity for school-age youth. J Pediatr.

[2] Ekelund U, Ja L, Sherar LB, Esliger DW, Griew P, Cooper A (2012). Moderate to vigorous physical activity and sedentary time and cardiometabolic risk factors in children and adolescents. JAMA.

[3] Department of Health. Start Active, Stay Active: A report on physical activity from the four home countries’ Chief Medical Officers. London: Department of Health; 2011.

[4] Cooper AR, Goodman A, Page AS, Sherar LB, Esliger DW, van Sluijs EM, Andersen LB, Anderssen S, Cardon G, Davey R (2015). Objectively measured physical activity and sedentary time in youth: the International children’s accelerometry database (ICAD). Int J Behav Nutr Phys Act.

[5] Jago R, Salway R, Emm-Collison L, Sebire SJ, Thompson JL, Lawlor DA (2019). Association of BMI category with change in children’s physical activity between ages 6 and 11 years: a longitudinal study (UNDER REVIEW).

[6] Metcalf B, Henley W, Wilkin T (2012). Effectiveness of intervention on physical activity of children: systematic review and meta-analysis of controlled trials with objectively measured outcomes (EarlyBird 54). BMJ.

[7] van Sluijs EM, McMinn AM, Griffin SJ (2007). Effectiveness of interventions to promote physical activity in children and adolescents: systematic review of controlled trials. BMJ.

[8] Atkin AJ, Gorely T, Biddle SJ, Cavill N, Foster C (2011). Interventions to promote physical activity in young people conducted in the hours immediately after school: a systematic review. Int J Behav Med.

[9] Riddoch CJ, Mattocks C, Deere K, Saunders J, Kirkby J, Tilling K, Leary SD, Blair SN, Ness AR (2007). Objective measurement of levels and patterns of physical activity. Arch Dis Child.

[10] Davies BR, Wood L, Banfield K, Edwards MJ, Jago R (2014). The provision of active after-school clubs for children in English primary schools: implications for increasing Children’s physical activity. Open J Prev Med.

[11] Jago R, Macdonald-Wallis C, Solomon-Moore E, Thompson JL, Lawlor DA, Sebire SJ (2017). Associations between participation in organised physical activity in the school or community outside school hours, and neighbourhood play with child physical activity and sedentary time: a cross-sectional analysis. BMJ Open.

[12] Beets MW, Beighle A, Erwin HE, Huberty JL (2009). After-school program impact on physical activity and fitness: a meta-analysis. Am J Prev Med.

[13] Beets MW, Glenn Weaver R, Brazendale K, Turner-McGrievy G, Saunders RP, Moore JB, Webster C, Khan M, Beighle A (2018). Statewide dissemination and implementation of physical activity standards in afterschool programs: two-year results. BMC Public Health.

[14] Mears R, Jago R (2016). Effectiveness of after-school interventions at increasing moderate-to-vigorous physical activity levels in 5- to 18-year olds: a systematic review and meta-analysis. Br J Sports Med.

[15] Jago R, Sebire SJ, Davies B, Wood L, Edwards MJ, Banfield K, Fox KR, Thompson JL, Powell JE, Mongomery AA (2014). Randomised feasibility trial of a teaching assistant led extracurricular physical activity intervention for 9 to 11 year olds: Action 3:30. Int J Behav Nutr Phys Act.

[16] Schuna J, Lauersdorf R, Behrens T, Liguori G, Liebert M (2013). An objective assessment of children’s physical activity during the keep it moving! After-school program. J Sch Health.

[17] Department of Transport. National Travel Survey: 2017. London: Department for Transport; 2018.

[18] McDonald NC (2007). Active transportation to school: trends among U.S. schoolchildren, 1969-2001. Am J Prev Med.

[19] Dollman J, Lewis NR (2007). Active transport to school as part of a broader habit of walking and cycling among south Australian youth. Pediatr Exerc Sci.

[20] Faulkner GE, Buliung RN, Flora PK, Fusco C (2009). Active school transport, physical activity levels and body weight of children and youth: a systematic review. Prev Med.

[21] Jago R, Edwards MJ, Sebire SJ, Tomkinson K, Bird EL, Banfield K, May T, Kesten JM, Cooper AR, Powell JE, Blair PS (2015). Effect and cost of an after-school dance programme on the physical activity of 11-12 year old girls: the Bristol girls dance project, a school-based cluster randomised controlled trial. Int J Behav Nutr Phys Act.

[22] Jago R, Sebire SJ, Wood L, Pool L, Zahra J, Thompson JL, Lawlor DA (2014). Associations between objectively assessed child and parental physical activity: a cross-sectional study of families with 5-6 year old children. BMC Public Health.

[23] Jago R, Bailey R (2001). Ethics and paediatric exercise science: issues and making a submission to a local ethics and research committee. J Sports Sci.

[24] Evenson KR, Catellier DJ, Gill K, Ondrak KS, McMurray RG (2008). Calibration of two objective measures of physical activity for children. J Sports Sci.

[25] Cole TJ, Freeman JV, Preece MA (1995). Body mass index reference curves for the UK, 1990. Arch Dis Child.

[26] Cole T. J (2000). Establishing a standard definition for child overweight and obesity worldwide: international survey. BMJ.

[27] Galobardes B, Shaw M, Lawlor DA, Lynch JW, Davey Smith G (2006). Indicators of socioeconomic position (part 1). J Epidemiol Community Health.

[28] Galobardes B, Shaw M, Lawlor DA, Lynch JW, Davey Smith G (2006). Indicators of socioeconomic position (part 2). J Epidemiol Community Health.

[29] StataCorp (2017). Stata Statistical Software: Release 15.

[30] Charlton C, Rasbash J, Browne WJ, Healy M, Cameron B. MLwiN Version 3.00. Centre for Multilevel Modelling. Bristol: University of Bristol; 2017.

[31] Leckie G, Charlton C (2013). runmlwin - a program to run the MLwiN multilevel modelling software from within Stata. J Stat Softw.

[32] Page A, Cooper AR, Griew P, Jago R (2010). Independent mobility, perceptions of the built environment and children’s participation in play, active travel and structured exercise and sport: the PEACH Project. Int J Behav Nutr Phys Act.

[33] Hansen BH, Anderssen S, Andersen LB, Hildebrand M, Kolle E, Steene-Johannessen J, Kriemler S, Page A, Puder JJ, Reilly JJ (2018). Cross-sectional associations of reallocating time between sedentary and active Behaviours on Cardiometabolic risk factors in young people: an international Children’s Accelerometry database (ICAD) analysis. Sports Med.

[34] Rutter H, Savona N, Glonti K, Bibby J, Cummins S, Finegood DT, Greaves F, Harper L, Hawe P, Moore L (2017). The need for a complex systems model of evidence for public health. Lancet.

[35] Sebire SJ, Edwards MJ, Campbell R, Jago R, Kipping R, Banfield K, Tomkinson K, Garfield K, Lyons RA, Simon J (2016). Protocol for a feasibility cluster randomised controlled trial of a peer-led school-based intervention to increase the physical activity of adolescent girls (PLAN-A). Pilot Feasibility Stud.

[36] Jago R, Salway R, Lawlor DA, Emm-Collison L, Heron J, Thompson JL, Sebire SJ. Profiles of children’s physical activity and sedentary behaviour between age 6 and 9: a latent profile and transition analysis. Int J Behav Nutr Phys Act. 2018;15:1-12. 10.1186/s12966-018-0735-8 PMC6199754 30352597

