# Supplementary material for: The association of school-related active travel and active after-school clubs with children’s physical activity: a cross-sectional study in 11-year-old UK children
Source: Int J Behav Nutr Phys Act. 2019 Aug 22;16:72. doi: 10.1186/s12966-019-0832-3 (PMC6704690; doi:10.1186/s12966-019-0832-3)
Supplement: Supplementary file 1 — Table S1. Comparison of sample characteristics with baseline. Table S2. Comparison of days with and without accelerometer measurements. Table S3. Associations of gender, SEP and BMI with participation in active after-school clubs. Table S4. Associations of gender, SEP and BMI with participation in active travel (from school). (DOCX 19 kb) [file 12966_2019_832_MOESM1_ESM.docx]

**Supplementary material for ‘The role of school-related active travel and active after-school clubs in children’s physical activity’**

Ruth Salway, Lydia Emm-Collison, Simon J. Sebire, Janice L. Thompson, Deborah A. Lawlor and Russell Jago

**Additional Tables**

Table S1: Comparison of sample characteristics with baseline

Table S2: Comparison of days with and without accelerometer measurements

Table S3: Associations of gender, SEP and BMI with participation in active after-school clubs

Table S4: Associations of gender, SEP and BMI with participation in active travel (from school)

Table S1: Comparison of sample characteristics with baseline

|  |  | Baseline (age 6) | Age 11 |
| --- | --- | --- | --- |
|  |  | mean (sd) or % | mean (sd) or % |
| Female | | 49% | 52% |
| Age | | 6.0 (0.4) | 11.0 (0.4) |
| IMD score | | 15.0 (12.9) | 15.4 (14.4) |
| Household education | |  |  |
|  | GCSE | 20% | 20% |
|  | A level | 28% | 26% |
|  | University degree | 36% | 37% |
|  | Higher degree | 16% | 17% |
| Baseline BMI z-score (at age 6) | | 0.27 (0.95) | 0.19 (0.92) |
| Baseline BMI category (at age 6) | |  |  |
|  | Healthy weight | 81% | 83% |
|  | Overweight | 11% | 12% |
|  | Obese | 8% | 5% |

Table S2: Comparison of days with and without accelerometer measurements

|  | non-accelerometer day | accelerometer day |
| --- | --- | --- |
| % attending active club | 15% | 15% |
| % active travel to school | 58% | 58% |
| % active travel from school | 60% | 59% |

Table S3: Associations of gender, SEP and BMI with participation in active after-school clubs

|  |  | | | OR | 95% CI | p-value |
| --- | --- | --- | --- | --- | --- | --- |
| **Gender** | | | |  |  |  |
|  | Male | | | 1 | Reference |  |
|  | Female | | | 0.99 | (0.86, 1.13) | 0.836 |
| **IMD** | | | | | |  |
|  | increase of 1 SD | | | 0.87 | (0.79, 0.96) | 0.005 |
| **Household education** | | | |  |  |  |
|  |  | | GCSE | 1 | Reference |  |
|  |  | | A level | 1.19 | (0.94, 1.50) |  |
|  |  | | University degree | 1.63 | (1.32, 2.03) |  |
|  |  | | Higher degree | 1.65 | (1.28, 2.11) | <0.0005 |
| **BMI category^1^** | | | | | |  |
|  |  | Healthy weight | | 1 | Reference |  |
|  |  | Overweight | | 1.18 | (0.95, 1.45) |  |
|  |  | Obese | | 1.12 | (0.90, 1.40) | 0.228 |
| **Weekend MVPA^2^** | | | |  |  |  |
|  | increase of 10 mins | | | 1.05 | (1.02, 1.08) | <0.0005 |

^1^ Model adjusted for confounders: gender, household education and IMD

^2^ Model adjusted for confounders: gender, household education, IMD and BMI category

Table S4: Associations of gender, SEP and BMI with participation in active travel (from school)

|  |  | | | OR | 95% CI | p-value |
| --- | --- | --- | --- | --- | --- | --- |
| **Gender** | | | |  |  |  |
|  | Male | | | 1 | Reference |  |
|  | Female | | | 0.67 | (0.61, 0.74) | <0.0005 |
| **IMD** | | | | | |  |
|  | increase of 1 SD | | | 0.79 | (0.74, 0.85) | <0.0005 |
| **Household education** | | | |  |  |  |
|  |  | | GCSE | 1 | Reference |  |
|  |  | | A level | 0.89 | (-0.76, 1.04) |  |
|  |  | | University degree | 1.19 | (1.03, 1.39) |  |
|  |  | | Higher degree | 1.16 | (0.97, 1.39) | <0.0005 |
| **BMI category^1^** | | | | | |  |
|  |  | Healthy weight | | 1 | Reference |  |
|  |  | Overweight | | 1.19 | (1.01, 1.40) |  |
|  |  | Obese | | 0.74 | (0.63, 0.87) | <0.0005 |
| **Weekend MVPA^2^** | | | |  |  |  |
|  | increase of 10 mins | | | 1.05 | (1.03, 1.08) | 0.020 |
| **Attends active club^3^** | | | |  |  |  |
|  | No | | | 1 | Reference |  |
|  | Yes | | | 1.19 | (1.00, 1.43) | 0.055 |

^1^ Model adjusted for confounders: gender, household education and IMD

^2^ Model adjusted for confounders: gender, household education, IMD and BMI category

^3^ Model adjusted for confounders: gender, household education, IMD, BMI category and weekend MVPA
